# “I can’t just talk it. I have to live it”: the roles, needs, and recruitment of recovery home house managers

**DOI:** 10.3389/fpubh.2026.1771917

**Published:** 2026-03-11

**Authors:** I. Niles Zoschke, Kathryn R. Gallardo, Hannah L. N. Stewart, Serena A. Rodriguez, Danielle Gillespie, Sheryl A. McCurdy, J. Michael Wilkerson

**Affiliations:** 1 Community Health Sciences Division, University of California at Berkeley, Berkeley, CA, United States; 2 Alcohol Research Group, Public Health Institute, Emeryville, CA, United States; 3 Department of Health Promotion and Behavioral Sciences, The University of Texas Health Science Center (UTHealth) at Houston School of Public Health, Houston, TX, United States; 4 Department of Health Promotion and Behavioral Sciences, The University of Texas Health Science Center (UTHealth) at Houston School of Public Health, Dallas, TX, United States

**Keywords:** medications for opioid use disorder, recovery support services, sober living homes, substance use disorder, workforce development

## Abstract

**Introduction:**

Recovery homes are an increasingly important recovery support service for people with substance use disorders, yet limited research has examined the roles of house managers or how individuals are recruited into these positions. This study examines how house managers understand and enact their roles, how recovery home operators recruit for these positions, and how the demands and sustainability of the role are perceived in practice.

**Methods:**

We conducted a qualitative thematic analysis of interviews with 29 operators and staff working in 10 Level II and Level III recovery homes across five Texas cities.

**Results:**

Findings indicate that house managers leverage lived experience to support residents, enforce house rules, and cultivate recovery-oriented house cultures that emphasize mutual accountability, peer engagement, and resident self-regulation. Operators most often recruited house managers from among successful residents with stable recovery and familiarity with house norms, though participants described limitations to this approach and noted that the role is frequently experienced as demanding and transitional.

**Discussion:**

These findings underscore the central role of house managers in shaping recovery home culture and highlight the complexity of a position that combines peer support, leadership, and operational responsibility within social model recovery settings. The results emphasize key balancing factors related to recruitment pathways, training, role sustainability, and length of employment. By documenting how house managers and operators conceptualize the role as both critical, yet often transitional, this study advances understanding of workforce dynamics in Level II and Level III recovery homes and identifies priorities for implementation-focused research on training, support structures, and staffing models that sustain recovery-oriented environments over time.

## Background

Recovery homes play a crucial role in addressing the needs of individuals recovering from a substance use disorder (SUD) by offering safe, socially supportive, drug free, communal living environments (1). The National Alliance for Recovery Residences (NARR) classifies recovery homes into four levels, Level I through Level IV, which differ by staffing, structure, and service provision (2). Level I residences are democratically run and peer led, with no paid staff. Level II residences employ staff who help manage operations and provide peer support. Level III residences similarly employ staff and additionally offer structured, nonclinical recovery support services. Level IV residences provide all of the above alongside licensed clinical services (2).

Across recovery housing levels, many homes are grounded in a social model of recovery that emphasizes peer learning, lived experience, mutual accountability, and the development of sober community as central mechanisms of change (3–5). In contrast to medical or professionalized treatment models, social model approaches prioritize experiential knowledge over credentials or formal clinical authority (3). Social model–based recovery homes frequently operationalize these principles by relying on peer recovery support staff, including house managers, who provide emotional support, practical assistance, education, and linkage to services grounded in lived experience (6). A growing body of evidence indicates that residence in recovery housing is associated with reduced substance use, lower risk of substance use recurrence, and improvements in employment, social functioning, and criminal justice outcomes (7–9). Within this context, lived experience and stability in recovery are widely regarded by practitioners as key sources of legitimacy and authority, while the emphasis on collective responsibility and resident self-governance raises important questions about how house managers balance leadership, accountability, and facilitation without undermining resident agency or democratic participation (4, 10–12).

Despite these benefits, recovery homes face persistent structural challenges, including limited and unstable funding, rising operational costs, and the ongoing risk of residents returning to substance use (13). These pressures are particularly salient for Level II and Level III recovery homes, which must maintain staffing, ensure compliance with certification standards, and provide consistent recovery support while operating within constrained financial environments (13). Within this context, house managers have emerged as central to the functioning, stability, and recovery orientation of Level II and Level III homes (14, 15).

House managers are typically responsible for overseeing daily operations, enforcing house rules, maintaining safety, and providing peer-based recovery support to residents (2). As the primary on-site staff in Level II and Level III recovery homes, they perform multiple roles, combining peer support with rule enforcement, mentorship, and administrative responsibilities, and prior quantitative research suggests they are instrumental in shaping house culture, promoting resident engagement, and supporting positive recovery outcomes (15). Consistent with social model principles, the role of house managers ideally extends beyond rule enforcement, role modeling, and individual support. In well-functioning recovery homes, house managers are expected to help cultivate environments in which residents themselves can grow in their recovery process (16). This function requires house managers to exercise judgment about when to intervene directly and when to allow residents to address challenges collectively. Although this balance is frequently discussed among recovery housing practitioners and operators, it has received limited systematic attention in the scholarly literature.

Much of the empirical literature on recovery housing focused on Level I or entirely peer-led homes, leaving significant gaps in understanding homes that employ on-site staff, such as house managers. What little has been written on recovery house managers has focused on discrete functions or outcomes rather than the lived experience of the role itself (15). Because house managers work and often live in Level II and Level III homes, which serve residents with more complex recovery needs, a more nuanced understanding of house manager roles, recruitment pathways, and workforce sustainability is urgently needed.

Existing research provides limited insight into how individuals become house managers or how recovery home operators recruit for these roles. Informal accounts suggest that operators often draw from within their own resident populations, promoting individuals who demonstrate stable recovery, leadership potential, and familiarity with house norms, yet recruitment strategies, selection criteria, and pathways into the house manager role remain largely undocumented in the peer-reviewed literature. Much of what is known about house managers instead resides in practitioner knowledge, organizational practice, and informal networks. Although recovery home operators, advocacy organizations, and house managers themselves often have well-developed understandings of effective leadership, recruitment, and role boundaries, these insights have not been systematically documented, theorized, or evaluated. As a result, policymakers, funders, and researchers lack a clear evidence base to inform staffing models, training strategies, and supports for Level II and Level III recovery homes, despite the central role staffing decisions play in shaping house culture, operational stability, and resident experiences.

Moreover, while house manager roles are often described by practitioners as demanding and emotionally taxing, there is limited empirical research examining the sustainability of the position over time, including turnover, role transitions, and career trajectories beyond recovery housing. Prior studies have noted that peer-based roles across substance use and recovery settings are frequently characterized by high expectations, a wide range of length of employment in their position, and varying degrees of social model principle implementation, and little formal training or education (15), yet little is known about how these dynamics specifically manifest within recovery homes or how they influence retention and workforce development.

The present study aims to describe the role of house managers in Level II and Level III recovery homes, to examine the challenges and difficulties associated with these positions, and to better understand pathways for recruiting and retaining individuals in these roles. By centering the perspectives of house managers and recovery home operators, this study addresses critical gaps in the recovery housing literature and generates practice relevant knowledge with direct implications for staffing, workforce development, and the delivery of recovery-oriented housing services.

## Methods

### Study design and parent study context

This qualitative analysis was conducted within Project HOMES (Housing for Opioid MAR Expanded Services), a multi-site mixed-methods evaluation of NARR-affiliate certified Level II and Level III recovery residences serving individuals taking medications for opioid use disorder (MOUD). A detailed description of the parent study protocol has been published previously (17).

### Setting

At the time of this analysis, the Project HOMES network included 15 recovery residences located in Austin, Houston, El Paso, Midland, and San Angelo, Texas. Eight homes served women and seven served men. Six residences were certified as NARR Level II and nine as NARR Level III.

All participating homes were required to accept residents taking any FDA-approved MOUD, including buprenorphine, methadone, and oral or injectable naltrexone. Each residence maintained formal written policies governing medication storage, dispensing procedures, and MOUD-related operational practices. Eight residences exclusively served individuals taking MOUD, whereas the remaining homes were mixed settings serving residents taking and not taking MOUD. Most homes were independent residential properties housing fewer than 15 residents; however, a few were integrated with SUD treatment facilities and also had more than 15 residents at any given time. All recovery residences involved in the study considered themselves as following social model principles in program implementation.

Homes were not randomly selected. Rather, they were identified through collaboration between the research team and the statewide NARR affiliate based on provider willingness to partner, operational capacity to open or transition Level II or III homes, geographic distribution across Texas, and the presence of local MOUD treatment infrastructure and recovery support services. As such, the participating residences represent a purposive network of community partners rather than a representative sample of recovery residences statewide.

### Study participants and sampling

Participants were recovery residence providers occupying operational and leadership roles within the participating homes. These roles included house managers responsible for day-to-day operations and peer support to residents, program directors overseeing programming and supervision, and operators or organizational leaders managing financing, certification, partnerships, and administrative functions.

Eligibility criteria required that participants be formally affiliated with a Project HOMES recovery residence as an operator, program director, or house manager, be 18 years of age or older, and be able to complete the interview in English or Spanish.

Sampling procedures differed across the two phases of data collection. During Phase 1 (October 2021 to May 2022), we used convenience sampling. Research staff conducted biannual site visits to participating recovery homes and invited eligible providers to participate in interviews. When feasible, we aimed to obtain representation from each participating residence and across operational roles. Twenty-nine providers representing 14 recovery homes participated during this phase; the fifteenth home joined the network after Phase 1 data collection concluded. In four instances, a second interview was conducted with the same participant during this period to obtain additional depth on topics that could not be fully explored during the initial interview.

During Phase 2 (June to July 2023), we re-contacted Phase 1 participants and used purposive sampling to conduct follow-up interviews focused on sustainability and longer-term operational considerations. Sampling goals for Phase 2 included maintaining representation across geographic sites and organizational roles and ensuring inclusion of providers from both male- and female-serving residences. Sixteen of the original 29 participants completed a follow-up interview.

See the Table 1 for sample characteristics, including city and profession, across both data collection phases. Demographic characteristics such as age, gender identity, race or ethnicity, and years of experience were not systematically collected from provider participants during qualitative interviews and therefore cannot be reported.

**Table 1:** Participant characteristics.

Phase 1 data collection		Phase 2 data collection
Total	*N* = 29	*N* = 16
	*N* (%)	*N* (%)
City		
Austin	8 (27.6%)	4 (25.5%)
Houston	7 (24.1%)	3 (18.8%)
Midland	5 (17.2%)	3 (18.8%)
El Paso	4 (13.8%)	3 (18.8%)
San Angelo	5 (17.3%)	3 (18.8%)
Profession^a^		
Owners/operators	4 (13.8%)	7 (37.3%)
House managers	18 (62.0%)	6 (31.3%)
Program director	8 (27.6%)	6 (31.3%)

a Does not add up to 100% because some participants had multiple roles.

### Data collection

Data were collected in two sequential phases (see the Study Participants and Sampling section for phase timelines). In Phase 1, semi-structured interviews focused on recovery home operations and participants’ experiences managing or working in homes that included residents taking MOUD as part of their recovery. Interview domains included house structure and policies, staffing and supervision, resident dynamics, implementation of MOUD-inclusive practices, and perceived facilitators and challenges associated with operating recovery residences. Interview guides were iteratively refined across phases to reflect emerging implementation themes and evolving operational priorities.

Approximately one year later, Phase 2 interviews were conducted to extend rather than repeat Phase 1 discussions. These interviews focused specifically on recovery home sustainability, including house policies, funding structures, partnerships, major expenses, resource acquisition, and factors influencing continued operation over time. Questions were framed around participants’ current experiences and the practical considerations affecting their ability to continue serving residents, including those taking MOUD.

Across both phases, interviews were conducted either in person during site visits or virtually using secure teleconferencing software. All interviews were audio-recorded with participant permission and professionally transcribed for analysis. Participants provided verbal informed consent prior to participation. No financial incentives were provided to provider participants.

### Data analysis

We used a pragmatic, applied qualitative analytic approach grounded in Saldaña’s provisional coding methods and iterative thematic analysis (18). The analysis was not guided by a formal theoretical framework; rather, interpretation was oriented toward understanding the experiences and perceptions of individuals working in recovery housing that support residents who take MOUD as part of their recovery. Provisional codes were developed based on the study research questions and qualitative interview guide questions.

The qualitative analysis team developed an initial deductive codebook based on the study research questions which was applied to the Phase 1 data. This provisional codebook was then tested against a subset of transcripts and refined through iterative discussion as the analysts became familiar with the data (19). Inductive, data-driven codes were added as new concepts and patterns emerged. Two analysts (INZ and HLNS) independently coded transcripts and met weekly to compare code application, discuss discrepancies, and revise the codebook. Coding disagreements were resolved through deliberation and negotiated consensus between the two analysts, with periodic peer debriefing sessions involving project leads (SAM, KRG) to review emerging interpretations and ensure analytic rigor and fidelity to the data (20, 21). Following refinement of the codebook, the same two analysts applied the coding framework across all Phase 1 transcripts. After Phase 2 interviews were completed, two qualitative analysts (INZ and DG) applied the final codebook from Phase 1 to the Phase 2 data.

Thematic analysis was conducted by identifying patterns, similarities, and differences across coded segments (22). Analysts developed analytic memos and provisional thematic statements throughout the coding process, drawing on observations recorded during data collection and analytic discussions. An example of a thematic statement is “A good manager should have lived experience, be rooted in recovery, be a peer to residents, and must know how to run a home. They also need to be good at solving problems.” One qualitative analyst (INZ; the first author) then consolidated and refined the analytical statements to develop themes. These memos supported theme development and served as a reflexive record of analytic decisions. Themes were iteratively refined through discussion between the two analysts and through peer debriefing with project investigators. Analytic adequacy was supported through iterative codebook development, independent coding followed by negotiated consensus, thematic saturation, reflexive memoing, and ongoing peer debriefing with senior investigators. These procedures enhanced credibility and consistency in interpretation across the dataset.

Researcher positionality was explicitly considered throughout the analytic process. Three members of the research team, including project leads who participated in peer debriefings, were involved in implementing Project HOMES and maintained working relationships with recovery residence operators. The team remained attentive to how this proximity could shape interpretation and participant disclosure. Reflexivity was addressed through regular discussion during analytic meetings, and confidentiality protections and data security procedures were emphasized during recruitment and consent to support candid participation.

## Results

### Sample characteristics

Interview participants were from Austin, Houston, Midland, El Paso, and San Angelo, Texas. There were a total of 7 operators, 18 house managers, and 8 program directors interviewed over both phases of data collection, with some individuals holding multiple titles within their recovery home.

### Thematic analysis

The first theme reveals that house managers were essential to recovery home operations. They leveraged lived experience to support residents, hold residents accountable, and build healthy house culture. The second theme describes how operators recruited ideal house managers by hiring successful residents and from external recovery and behavioral health organizations. The third theme demonstrates that the manager role is difficult personally and professionally, and eventually house managers move on to different roles and endeavors.

#### House manager roles and responsibilities

Unanimously, participants believed that without good house managers, a recovery home could not function. House managers were responsible for many tasks, including running house meetings, managing the budget, maintaining NARR-affiliate certification, purchasing supplies, mentoring residents, and enforcing rules. Much of their responsibility, however, involved working directly with residents to support their recovery. Operators and house managers emphasized the advantages of Level II and Level III recovery homes over Level I homes because of the increased accountability and support house managers provide to residents. Due to the many roles they filled, house managers felt they had to balance being a peer role model and a rules enforcer. This was a delicate matter and required subtle approaches, like building trust with residents during one-on-one time. One house manager said about striking that balance:

Sometimes guys will come in kind of broken in need of someone to...to sit, like to be there for them. Not, I’m not saying like where I’m crossing, I’m not the emotional support, right but more of like, you know, they need someone to talk to. It's uncomfortable. They don't know where they fit in. So, I may be there to guide them. We've gone on meetings with guys. I’ve gone on walks with guys. Someone come [sic] and hung out in my room. We'll listen to music. But, you know, certainly, I’ve noticed that like I’ll be kind of, kind of close in a way, you know, how like a normal friend, almost a friendship would develop. So, there's kind of that build up. There's some bond and then I kind of like let them go, you know, as they integrate in the house.

-House manager

According to operators, directors, and house managers, a good house manager needed lived experience to be a relatable role model. Residents respected, and were more willing to accept guidance from, house managers with lived experience. Compassion and being judgment-free were also seen as key because house managers had to distinguish when a resident needed accountability for disrespecting the house rules or grace for making mistakes. “Compassion along with boundaries. They go hand in hand,” said one program director on the role of a house manager. House managers also needed to be good at solving problems to keep the houses running. One operator remarked on the essential roles of a house manager:

It's a big role. They're there all the time. They have to be a positive role model. They have to exhibit, you know, their own positive lifestyle behaviors, uh, healthy lifestyle behaviors. Uh, they will really help us set the tone for enforcing our rules and our structure. If...if we have someone that does not observe the standards that we, that we hold to, we're really not helping that person in recovery… And we believe in growing the individual not just in recovery but as a person. And that house manager is key to that. Because if they don't support that and promote that and enforce that, then we are pretending, not really delivering a good program.

-Operator

House managers established house culture to promote residents’ recovery and set the tone for the home. The notion of house culture was closely related to and influenced by the social model of recovery, involving peer learning, social engagement, and social support between residents. Operators described a healthy house culture as involving residents refraining from stigmatizing behavior, respecting one another, having a mindset focused on growing in recovery, and holding each other accountable for following house rules. Sometimes house managers described house culture as being part of a family. To set a healthy tone for homes, house managers led weekly house meetings emphasizing mutual accountability, taking time to address disputes between residents. They also led morning meditation daily to start the day with positive affirmations and a focus on achieving recovery goals. Furthermore, house managers modelled healthy habits and life skills by demonstrating consistent leadership, even-temperedness, and embodying the outcomes of successful recovery. Healthy house cultures enabled strong communication, openness, honesty, compassion, accountability, and growth.

Modeling healthy recovery, maintaining peer-relatability, and holding residents accountable to house rules came with inherent tensions, though. House managers and program directors described that residents did not always like being pushed even if it ultimately helped them grow in recovery. Sometimes operators found managers drifting from the rules and had to reestablish appropriate boundaries and guidelines, including randomly drug testing house managers alongside residents. One program director called balancing house manager peer leadership with holding residents accountable to house rules as a “yin and yang.”

There's the yin and yang of house managers’ role… There's the accountability portion that is always balanced by the social model, right? Like being a peer [is] essentially a member that helps organize almost like a family environment. And so that person has to be able to balance that with the accountability figure that says, you know, "You do have to be home by curfew. You do have to do your chores." We try not to take an authoritative approach, but of course, there has to be, like, some, uh, accountability parameters for that, like, family environment to live with it.”

-Program Director

Social support, mutual respect, and group dedication to recovery helped residents stay on track and get back on track if they socially withdrew or fell into bad habits. Participants across the board warned that one disengaged or negative resident could threaten the entire house, and house managers correcting these issues was important for the entire program. This was challenging for house managers, especially when they had to discharge a resident who returned to use. One house manager remarked on the difficulties with emotionally connecting with at-risk residents while working on his own recovery:

I can't let an individual that returns to use and puts the safety of other residents, you know, I can't let them stay no matter, no matter if I’m fond of them or not. And being fond of people, I can't show favoritism either. But there has to be an understanding of ethical guidelines and there has to be shown. And it's not just whatever, it’s like I have to live it, right, like I can't just talk it. I have to live it.

-House manager

House managers took the safety and success of residents seriously. The importance of a house manager’s role in a recovery home was crucial because of the many different roles residence managers played, and how vulnerable residents often were. Managers were a lifeline for residents. “We support them all,” said one house manager. To many house managers, their job was also a calling. Illustrating this point, a house manager said:

…I think you have to have a passion for it, because it can get very frustrating, so you have to have a passion for wanting to help people. I can’t imagine doing anything else… I love my job. Even when I’m about to pull out my hair, I love my job.

-House manager

Taken together these sentiments and quotations illustrate that keeping recovery home operations going requires great house managers with many roles, skills, and qualities, ranging from functional attributes to cultural qualities, and having lived experience. This unique set of requirements made house management a difficult balancing act of peer-relatability and authority. It also made the ideal managerial candidate a very specific person.

#### Recruiting ideal house managers

House managers had to exhibit specific qualities and be willing to do a demanding job. They were hard to recruit. To find candidates with lived experience, compassion, and a sense of recovery home culture, operators and program directors often relied on recruiting house managers from former residents. Residents-turned-managers benefited from stable employment while maintaining housing, learning new skills, and giving back to the recovery community from which they benefited. They also leveraged the opportunity to achieve their own long-term recovery goals. One operator said of recruiting from a current resident:

I prefer for them [house managers] to raise up through the ranks so to speak. I prefer them to be residents, resident advisor, manager. Because they know the culture, they know the system… And they are a product of it. And if they’ve been around for any amount of time and bought into it, their lives are improved because of it, and they have an intimate working knowledge of it. Their lives are improved because of it, and therefore they advocate for it. They’re like, “Yeah, this is the way to do things.”

-Operator

Hiring a current resident as a house manager preserved a house’s culture and made things easier for operators because these candidates already knew how the house functioned. This practice also preserved relationships and trust within the homes for the residents and leveraged the strong bonds already built to a healthy home. These bonds helped other staff in the residences as well. One house manager said about her assistant manager who was hired as a resident, “She is like my saving grace.” She went on to say:

She is someone that the other women in the house look up to and so I wanna celebrate that and I want to reward it. I did at one point think what if we made this a temporary sort of position… so that other people could be celebrated and have this like chance to save while they're here and make a little money. But, um, at this point, I don't have anyone that I could do that with.

-House manager

While hiring a current resident to manage a level II or III home was an ideal strategy, it was not always possible. Some operators hired people from familiar 12-step groups or from other recovery communities, prioritizing those people for their lived experience. Other times, operators hired staff who had been employed by treatment facilities, but those candidates often espoused a “medical model” of recovery which does not apply elements of the social model of recovery. Thus, operators often retrained outside hires in social model principles and encouraged them to be open minded about many pathways to recovery. Operators also hired people outside the field of recovery, but preferred candidates with experience working in health or criminal justice institutions, and looked out for candidates with compassion, patience, and relevant skills. They also looked for candidates with substance use disorder lived experience and dedicated recovery, as house managers were expected to maintain sobriety.

#### Being a house manager is difficult personally and professionally

Many managers derived great satisfaction from supporting residents’ successes, but emotional fulfillment only took them so far. House managers struggled with unplugging from an emotionally demanding career. While not technically always on the clock, house managers often did not leave their worksite because they lived where they worked. Managers responded to emergencies at all times of day. A house manager remarked on the topic:

I guess physically… because you're in a community environment 24/7 and being a house manager… it’s really a difficult time to just walk through the house without, "Hey. Hey, this. Hey, this, hey this". And so, and you try to have those boundaries as much as possible like even letting them know like, "Hey, man, you know I’m not on clock time…”

-House manager

This quotation illustrates how important boundaries were for managers so they could have some time to themselves, even while living where they worked.

Managers also described self-care as an essential practice to cope with stress. It also helped them maintain the stamina to help residents day after day. One manager said, “I told my girls [residents] in our house meeting yesterday, ‘I’m concentrating on self-care this week because that is the dimension of recovery that is gonna affect all the rest of mine.’” Another house manager remarked:

Everything in this field revolves around mental health so it's like, you know, how can the residents get all the help they need? And then it's like, you know, God forbid a house manager needs to do something to take care of themselves, you know, obviously we have to...we can't help other people if we can't even help ourself [sic].

-House manager

Knowing the demands of the house manager role, recovery home operators strongly supported managers’ self-care. One house manager remarked, “The organization… they’re really big on our self-care, that, you know. They are because it’s, uh, you can face real burnout doing this job.” Taken in context, these quotations highlight the difficulties house managers face while living at work and steps they took to mitigate potential burnout from the role.

Because of the incredible demands and specific qualities house managers require, operators grappled with paying them enough to retain quality staff on thin budgets. One operator said:

“If I was gonna advise someone to do what we do, I would say, you know, ‘You have to get some well-trained recovery coaches with a good foundation in personal recovery,’ and, you know, that's an expense. And that's gonna become a greater expense as time goes on because these guys are gonna want better pay.”

-Operator

This participant said that without funding for full-time managers, house managers would need to find part-time work elsewhere. If recovery homes went to a part-time house manager model, service quality might decline.

House managers eventually moved on from their position. Operators and house managers described that as house managers advanced in their recovery, leadership, and personal lives, many factors led them to find other opportunities. Many expressed a desire for career advancement and continued involvement in the recovery community, even after leaving their jobs as house managers. Some wanted more autonomy, to earn higher wages, or eventually open their own recovery homes. Others hoped for more time and space to focus on personal goals, such as buying a home or spending more time with family and partners. In this way, living in a recovery home assisted a person in early and unstable recovery from substance use to stabilize their lives, get a rewarding job in-house, help lead and mentor others, achieve long-term personal recovery goals, and ultimately move on to make a big impact in the next phase of their commitment to the recovery community.

## Discussion

This analysis extends prior quantitative work on house managers in recovery housing by offering a qualitative examination of how house managers understand and enact their roles, how operators recruit for these positions, and how managers experience the demands and limits of the work. The study was nested within a larger mixed-methods evaluation, which created an opportunity to examine the perspectives, needs, and operational realities of peer recovery workers themselves using data collected as part of ongoing implementation research. While the importance of house managers to recovery home functioning and resident outcomes has been previously documented (15), the lived realities of the role, including pathways into the position and expectations regarding its duration, have remained underexamined. By foregrounding the perspectives of house managers and operators, this study clarifies how staffing practices shape the day to day operation of Level II and Level III recovery homes and illuminates gaps between social model ideals and organizational constraints.

Consistent with practitioner accounts but unexplored in scholarly literature, operators in this study most often promoted successful residents to the role of house manager in order to leverage lived experience, demonstrated sobriety, and familiarity with house lifeways. These recruitment strategies reflect social model principles that privilege experiential knowledge and peer legitimacy over formal credentials. However, participants also described limitations to this approach, noting that ideal resident candidates were not always available or willing to assume the role. In such cases, operators recruited peer recovery support specialists or healthcare workers from related settings. While these hires brought relevant experience, participants emphasized that individuals trained in medical or clinical models of care often required additional education to effectively apply social model principles within recovery homes.

These findings align with prior literature emphasizing the need for social model–specific training among peer recovery support specialists (12) and reinforce reports that recovery home staff desire guidance on developing house manager training materials (23). Importantly, this study does not evaluate the effectiveness of any particular training approach, but it does highlight a mismatch between the complexity of the house manager role and the limited availability of formal preparation or ongoing support. These gaps may help explain the variability in how house managers enact their roles across recovery homes. Although NARR standards emphasize professional development (10), NARR does not currently mandate or provide standardized training curricula for recovery home staff (24). Future research should systematically document how operators train house managers in practice and evaluate how different training models support the enactment of social model principles without undermining peer-based authority. Recovery advocacy organizations and community groups should develop house manager training materials, inspired by this research and existing work, like the Texas Recovery-Oriented Housing Network’s house manager training program (25).

Beyond recruitment and training, participants described the house manager role as requiring constant negotiation between peer support and rule enforcement. House managers were expected to provide experience-based guidance and emotional support while also maintaining structure, accountability, and safety within the home. Participants emphasized that deciding when to act as a peer and when to act as an authority figure was a balancing act. Prior research suggests that consistent rule enforcement is particularly important in recovery homes serving residents with criminal justice involvement, where even handed accountability can support conflict resolution and collective responsibility (26). At the same time, residents have described house managers’ encouragement and motivation as a critical source of support, reinforcing the need for a balance between accountability and care (26). Taken together, these findings suggest that the optimal balance of peer support and enforcement may vary depending on resident characteristics and home structure. This study illustrates how house managers actively mediate these principles in practice, often under conditions of limited guidance and high expectations. The findings do not suggest a single ideal approach but rather underscore the situational and relational nature of leadership in staffed recovery homes.

In addition to mediating peer support and rule enforcement, findings from this study highlight house managers’ central role in actively establishing and sustaining house culture as a mechanism for resident recovery. Participants consistently described house culture as closely aligned with social model principles, emphasizing peer learning, mutual accountability, social engagement, and shared responsibility for maintaining a recovery-oriented environment. A healthy house culture was characterized by residents refraining from stigmatizing behavior, demonstrating respect for one another, maintaining a mindset oriented toward growth in recovery, and holding one another accountable for following house rules. In some cases, participants explicitly described this culture using familial language, underscoring the relational and affective dimensions of recovery housing.

House managers were described as intentionally setting the tone for the home through concrete, routine practices rather than relying solely on formal authority. These practices included leading weekly house meetings that emphasized mutual accountability and provided structured opportunities to address interpersonal conflict, as well as facilitating daily morning meditation to orient residents toward recovery goals and positive affirmations. Beyond structured activities, house managers were also described as modeling healthy habits and life skills through consistent leadership, emotional regulation, and even tempered responses to challenges, thereby embodying the outcomes of successful recovery. Participants viewed these behaviors as critical to fostering environments characterized by openness, honesty, compassion, and accountability, which in turn supported residents’ ongoing recovery processes. Together, these practices positioned house managers as facilitators of resident self-regulation rather than sole enforcers of compliance. This finding extends the recovery housing literature by illustrating how house managers contribute to recovery not only through rule enforcement or individual support, but by enabling residents to hold themselves and one another accountable within a shared cultural framework.

A central contribution of this study is its documentation of how house managers and stakeholders understand the temporality of the role. Participants consistently described the house manager position as demanding, emotionally taxing, and difficult to sustain indefinitely. Although many house managers found the work meaningful and enriching to their own recovery, they also reported working long hours, making personal sacrifices, and experiencing ongoing stress. As a result, both house managers and operators often viewed the position as a transitional role, one that allowed individuals to give back to the recovery community, strengthen their own recovery, and develop skills before moving on to other opportunities. To assist with the costs of professional development and training, formalized career advancement structures should be supported by state or federal funding. Advocacy organizations, state agencies, and academic partners must work proactively to support house managers in their current positions and for their next career steps and personal growth.

From a workforce development perspective, this finding raises important questions about house manager length of employment. Prior research documents substantial variability in house manager tenure, with reported durations ranging from weeks to many years, reflecting the absence of a standardized or normative career trajectory for the role (15). Although prior studies do not directly examine house manager tenure, research suggests that stable and supportive recovery home social environments are associated with longer resident retention, implying that continuity of leadership may play an important role in shaping these conditions (8, 11). At the same time, shorter or transitional roles may align more closely with social model values by preventing over-professionalization, preserving peer relatability, and allowing individuals to cycle through leadership as part of their recovery journey. This study does not determine the optimal duration of house manager tenure, but it does suggest that recovery housing organizations should intentionally consider these tradeoffs rather than treating turnover as either a failure or an inevitability, and design staffing models that support both effective short term leadership and sustainable longer term roles depending on house context and resident composition.

Importantly, while participants expressed interest in career advancement within recovery advocacy and related fields, the study does not provide evidence on whether such pathways improve outcomes for residents or staff. Participants said that organizational support and time off for self-care were essential strategies for mitigating burnout. Our results suggest that higher wages and access to professional development might improve working conditions and potentially extend tenure. Future research should examine how different staffing models, compensation structures, and career ladders influence both house manager sustainability and recovery home functioning over time.

Consistent with participants’ emphasis on experiential legitimacy and peer relatability, practitioners may need to remain cautious against uncritical professionalization of the house manager role. Historical analyses of social model recovery note that increased credentialing and formalization could risk distancing peer recovery support specialists from the people they serve and erode the experiential legitimacy that underpins peer-based recovery support (27). While professional development may address training gaps and workforce instability, practitioners and advocates need to balance such efforts with the preservation of social model principles. Participatory research approaches that involve house managers in the design of training, support structures, and career pathways may help navigate these tensions while centering practitioner expertise.

### Limitations

Several limitations should be considered when interpreting these findings. First, the data may be subject to social desirability bias, as the researchers who conducted the interviews were affiliated with the parent study that funds the recovery homes included in the analysis. Although participants were encouraged to respond candidly and assured of confidentiality, some may have moderated their responses. Second, the sample was limited to a relatively small number of recovery homes within a single state and relied on voluntary participation, which may have introduced self-selection bias and limits representativeness. Participant demographic characteristics beyond job title and city were not systematically collected, limiting our ability to assess how perspectives may have varied by factors such as gender, age, or professional background. Third, findings are based on self-reported qualitative data from operators and staff and do not include direct observation, resident perspectives, or linkage to quantitative outcomes, precluding causal inference regarding the effects of specific house manager practices or staffing models. Fourth, while systematic qualitative analytic procedures were used, theme development necessarily involved interpretive judgment, and alternative analytic approaches may have emphasized different aspects of the data. Finally, recovery homes vary widely by level, structure, resident population, and policy context; therefore, findings are most transferable to Level II and Level III recovery homes operating within similar social model frameworks and should be applied cautiously to other settings. Because all participating residences supported residents who take MOUD as part of their recovery, findings may not generalize to recovery homes that prohibit or restrict MOUD or operate under abstinence-only models. In addition, this analysis combined Level II and Level III homes, which differ in staffing structure and service intensity; although this reflects real-world implementation within the Project HOMES network, it may obscure differences in managerial roles and expectations across levels. Despite these limitations, the study provides contextually grounded insights into an underexamined area of recovery housing research and offers a foundation for future work incorporating resident perspectives, longitudinal designs, and mixed-methods approaches.

## Conclusion

In conclusion, this study leverages recovery home staff perspectives to provide a qualitative examination of the roles house managers play in Level II and Level III recovery homes, how individuals are recruited into these positions, and how the demands and limits of the role are understood in practice. Findings highlight house managers’ contributions not only to rule enforcement and individual support, but also to the cultivation of recovery-oriented house cultures that promote mutual accountability, peer engagement, and resident self-regulation. Importantly, this analysis documents that recovery home staff frequently conceptualize the house manager position as a demanding and often transitional role, one that can meaningfully support residents while also contributing to house managers’ own recovery and professional development. Rather than prescribing an ideal staffing model, the findings underscore the need for recovery housing organizations to intentionally consider tradeoffs related to training, role sustainability, and length of employment, while preserving core social model principles. This study also demonstrates the value of embedding workforce-focused inquiry within larger implementation research efforts, suggesting that future studies of recovery services should similarly leverage ongoing evaluations to examine the dynamics, needs, and experiences of the peer recovery workforce itself. These findings have implications for recovery home operators and advocacy organizations and underscore the need for future research that incorporates participatory methods, attends to workforce development trajectories, and assesses the role of staffing models in shaping recovery home functioning over time.

## Data Availability

The original data applied in this research cannot be distributed publicly because the data may be personally identifiable. Requests to access the datasets should be directed to sheryl.a.mccurdy@uth.tmc.edu.

## References

[1] Mericle AA Miles J Way F. Recovery residences and providing safe and supportive housing for individuals overcoming addiction. J Drug Issues. (2015) 45:368–84. doi: 10.1177/0022042615602924

[2] NARR. Recovery residence levels of support (2016). Available online at: https://narronline.org/wp-content/uploads/2016/12/NARR_levels_summary.pdf

[3] Borkman T. Resident self-governance in social model recovery programs. Contemp Drug Probl. (1998) 25:741–71. doi: 10.1177/009145099802500405

[4] Borkman TJ Kaskutas LA Room J Bryan K Barrows D. An historical and developmental analysis of social model programs. J Subst Abus Treat. (1998) 15:7–17. doi: 10.1016/S0740-5472(97)00244-4, 9534122

[5] Wright A. "What is a social model?". In: Shaw S Borkman T, editors. Social Model Alcohol Recovery: An Environmental Approach. Burbank, CA: Bridge-Focus, Inc. (1990). p. 7–10.

[6] Solomon P. Peer support/peer provided services underlying processes, benefits, and critical ingredients. Psychiatr Rehabil J. (2004) 27:392–401. doi: 10.2975/27.2004.392.401, 15222150

[7] Jason LA Olson BD Ferrari JR Majer JM Alvarez J Stout J. An examination of main and interactive effects of substance abuse recovery housing on multiple indicators of adjustment. Addiction. (2007) 102:1114–21. doi: 10.1111/j.1360-0443.2007.01846.x, 17567399 PMC2976482

[8] Jason LA Guerrero M Salomon-Amend M Stevens E Light JM Stoolmiller M. Context matters: home-level but not individual-level recovery social capital predicts residents’ relapse. Am J Community Psychol. (2021) 67:392–404. doi: 10.1002/ajcp.12481, 33296504 PMC9149681

[9] Polcin DL Korcha RA Bond J Galloway G. Sober living houses for alcohol and drug dependence: 18-month outcomes. J Subst Abus Treat. (2010) 38:356–65. doi: 10.1016/j.jsat.2010.02.003, 20299175 PMC2860009

[10] NARR. National Standard 3.0 compendium (2018). Available online at: https://narronline.org/wp-content/uploads/2024/05/NARR-National-Standard-3.0-Compendium.pdf

[11] Mahoney E Witbrodt J Mericle AA Polcin DL. Resident and house manager perceptions of social environments in sober living houses: associations with length of stay. J Community Psychol. (2021) 49:2959–71. doi: 10.1002/jcop.22620, 34076263 PMC8380640

[12] Mericle AA Howell J Borkman T Subbaraman MS Sanders BF Polcin DL. Social model recovery and recovery housing. Addict Res Theory. (2023) 31:370–7. doi: 10.1080/16066359.2023.2179996, 37928886 PMC10624396

[13] Zoschke IN Gallardo KR Gillespie D Stewart HL Rodriguez SA McCurdy SA . Sustaining substance use recovery housing for people taking medications for opioid use disorder: diverse funding, strategic partnerships, and charging rent. Front Public Health. (2025) 13:1528971. doi: 10.3389/fpubh.2025.1528971, 40371296 PMC12075534

[14] Mericle AA Polcin DL Hemberg J Miles J. Recovery housing: evolving models to address resident needs. J Psychoactive Drugs. (2017) 49:352–61. doi: 10.1080/02791072.2017.1342154, 28657823 PMC5998815

[15] Polcin DL Mahoney E Mericle AA. House manager roles in sober living houses. J Subst Abus. (2021) 26:151–5. doi: 10.1080/14659891.2020.1789230, 33732093 PMC7958703

[16] Gallardo KR Zoschke IN Stewart HL Wilkerson JM Henry EA McCurdy SA. Supporting medication-assisted recovery in recovery residences: staff support, managing built environment threats, and building a supportive network. Am J Drug Alcohol Abuse. (2024) 50:739–47. doi: 10.1080/00952990.2024.2401983, 39382549

[17] Wilkerson JM Gallardo KR Rodriguez S Brown HS Cazaban CM Yang JJ . Expansion and evaluation of level II and III recovery residences for people taking medications for an opioid use disorder: project HOMES (housing for MAR expanded services) study protocol. BMJ Open. (2024) 14:e084115. doi: 10.1136/bmjopen-2024-084115, 39496371 PMC11535685

[18] Saldaña J. The Coding Manual for Qualitative Researchers.SAGE Publications Ltd (2021).

[19] Nowell LS Norris JM White DE Moules NJ. Thematic analysis: striving to meet the trustworthiness criteria. Int J Qual Methods. (2017) 16:1609406917733847. doi: 10.1177/1609406917733847

[20] Roberts K Dowell A Nie JB. Attempting rigour and replicability in thematic analysis of qualitative research data; a case study of codebook development. BMC Med Res Methodol. (2019) 19:1–8. doi: 10.1186/s12874-019-0707-y, 30922220 PMC6437927

[21] Sandelowski M Sandelowski MJ Barroso J. Handbook for Synthesizing Qualitative Research.Springer Publishing Company (2007).

[22] Tolley EE. Qualitative Methods in Public Health: A Field Guide for Applied Research.Wiley (2016).

[23] Miles J Bunn T Kizewski A Jennings T Waters T Johnson D . Assessing technical assistance needs among recovery residence operators in the United States. J Psychoactive Drugs. (2022) 54:188–95. doi: 10.1080/02791072.2021.1941442, 34269163

[24] NARR. MAT-Capable Recovery Residences: How State Policymakers Can Enhance and Expand Capacity to Adequately Support Medication Assisted Recovery. St Paul, MN: National Alliance for Recovery Residences (2018). p. 1–17.

[25] Texas Recovery-Oriented Housing Network. Course catalog. (2025). Available online at: https://trohn.org/edu/

[26] DeGuzman R Korcha R Polcin D. “I have more support around me to be able to change”: a qualitative exploration of probationers’ and parolees’ experiences living in sober living houses. Int. J. Ther. Communities. (2019) 40:51–65. doi: 10.1108/TC-04-2018-0008, 31467467 PMC6715296

[27] Borkman T Kaskutas LA Owen P. Contrasting and converging philosophies of three models of alcohol/other drugs treatment: Minnesota model, social model, and addiction therapeutic communities. Alcohol Treat Q. (2007) 25:21–38. doi: 10.1300/j020v25n03_03

